# Dermatomyositis as a Paraneoplastic Syndrome in Cases of Aggressive Cervical Cancer

**DOI:** 10.7759/cureus.79838

**Published:** 2025-02-28

**Authors:** Kandace Williams, Maria Carmona-Gonzalez

**Affiliations:** 1 Internal Medicine, University of Florida College of Medicine, Pensacola, USA

**Keywords:** adult-onset dermatomyositis, advanced cervical cancer, cancer screening strategy, cervical carcinoma, paraneoplastic dermatomyositis

## Abstract

Dermatomyositis is an inflammatory myopathy characterized by proximal muscle weakness with concurrent cutaneous manifestations. Diagnosis is often made by the clinical presentation in conjunction with elevated muscle enzymes, serology, electromyogram findings, and muscle biopsy. Dermatomyositis is frequently complicated due to its known correlation with underlying malignancy, even if not diagnosed at the time of presentation. Timely treatment and screening strategies can help to improve patient outcomes and both quality and quantity of life. In this study, we describe a female diagnosed with dermatomyositis who unfortunately had rapid progression and development of invasive squamous cell carcinoma of the cervix.

## Introduction

Dermatomyositis (DM) is an idiopathic autoimmune connective tissue disease often characterized by its cutaneous manifestations. The prevalence of DM is approximately 13 per 100,000 people and tends to affect females more commonly [1]. The risk of DM tends to increase with age [1]. The most common presenting symptoms of DM include progressive proximal muscle weakness with concurrent skin findings; however, cutaneous manifestations may be the only presenting symptom and linger long after completion of treatment [1,2]. Pathognomonic cutaneous findings include the classic Gottron's papules on the hands, heliotrope periorbital eruption, and several other pruritic, erythematous eruptions in photo-distributed areas such as the scalp, neck, extensor surfaces, chest, and back [2].

Many patients with dermatomyositis unfortunately have a significant impact on their quality of life due to this disease. Mainly due to intense pruritus that can progress to even burning paresthesia [2]. Patients with classic DM can also present with proximal muscle weakness which can progress to dysphagia and even dyspnea. This unrelenting disease course often leads to progressive fatigue, weight loss, and chronic debilitation [1-3].

There is a well-known association between the diagnosis of DM and underlying malignancy. It has been documented to have a six-fold higher risk of the development of malignancy compared to the general population [4]. Oftentimes, DM is the presenting symptom in patients who are unaware of an underlying malignancy. This poses a unique perspective in the continued screening recommendations of these patients once diagnosed.

In this study, we will discuss a case of classic dermatomyositis with an unfortunate unfolding of disease progression along with an associated diagnosis of invasive cervical cancer. We will then discuss current malignancy screening guidelines to help educate others on the importance of both prompt recognition of this disease state and early screening strategies.

## Case presentation

**Figure 1:**
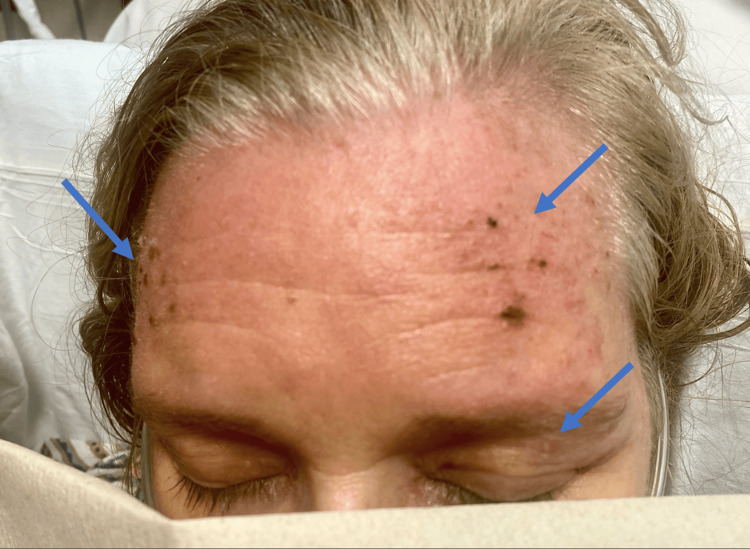
Heliotrope rash (arrows) over the eyelids and forehead of the patient with dermatomyositis.

**Figure 2:**
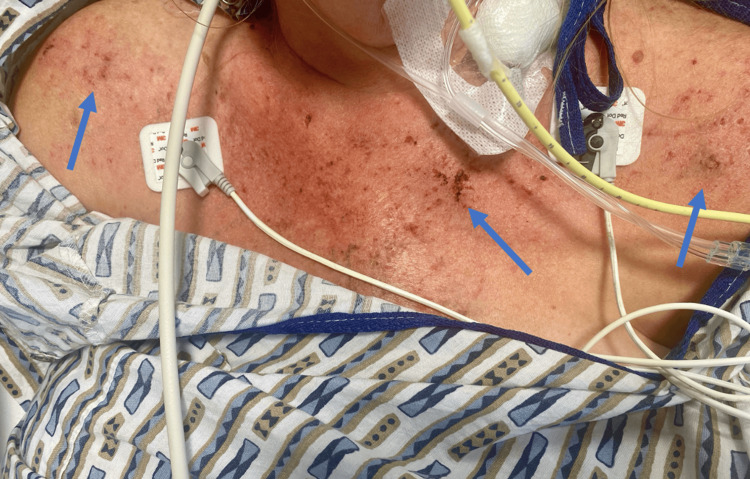
“V-sign” (arrows) chest rash in a patient with dermatomyositis.

**Figure 3:**
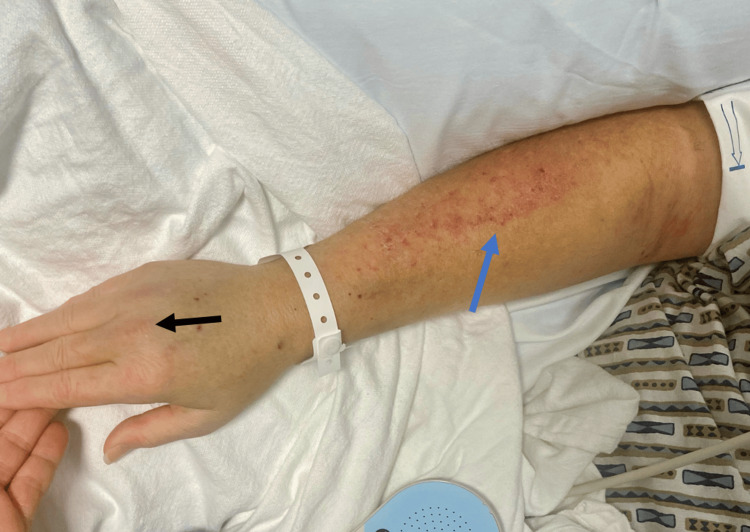
“Sleeve sign” (blue arrow) with healing Gottron’s papules (black arrow) in the patient with dermatomyositis.

A 55-year-old woman with a medical history of untreated hypertension and newly diagnosed type 2 diabetes mellitus presented to the hospital due to four weeks of progressive myalgias, an erythematous rash, and signs of an upper respiratory infection. She reported a productive cough, weakness with difficulty transitioning from sitting to standing, and a pruritic rash on her face, scalp, chest, back, and anterior thighs (Figures 1-3).

Initial vital signs demonstrated sinus tachycardia with a heart rate of 135 beats per minute, respiratory rate of 18 breaths per minute, blood pressure of 173/133 mmHg, temperature of 36.6°C, and oxygen saturation of 96% on ambient air. The patient was subsequently found to have low-risk pulmonary emboli (PE) and a right pleural effusion on computed tomography angiography (CTA) of the chest in addition to her rash concerning dermatomyositis. She was placed on therapeutic enoxaparin 1 mg/kg every 12 hours for treatment of her PE.

Hematologic evaluation demonstrated leukocytosis with neutrophilia. Inflammatory markers including erythrocyte sedimentation rate and C-reactive protein were both normal. Other hematologic findings are noted in Table 1. Biochemical findings included elevated alanine aminotransferase and aspartate aminotransferase, creatinine kinase, and lactic acid. Please refer to the values presented in Table 2. Urine analysis did note evidence of proteinuria, glycosuria, and the presence of blood without red blood cells. Urine drug screen, hepatitis panel, thyroid studies, and HIV assay were negative.

**Table 1:** Initial hematologic evaluation of a patient with dermatomyositis. ^*^Abnormal lab values.

Parameter	Result	Reference range
Red blood cells	6.2 million cells/μL^*^	3.8-5.2 million cells/μL
Hemoglobin	17.3 g/dL^*^	12.0-15.4 g/dL
Hematocrit	53.2%^*^	35-45%
White blood count	11.8 thousand cells/μL^*^	4.0-11.0 thousand cells/μL
Neutrophils	84.1%^*^	40-70%
Lymphocytes	4.8%	20-45%
Eosinophils	0.0%	1-6%
Monocytes	9.8%	2-10%
Basophils	0.4%	0-1%
Platelet count	255 thousand cells/μL	150-400 thousand cells/μL
ESR	8 mm/h	<20 mm/h

**Table 2:** Initial biochemical evaluation of a patient with dermatomyositis. ^*^Abnormal lab values.

Parameter	Result	Reference range
Renal function tests		
Sodium	129 mmol/L^*^	136-145 mmol/L
Potassium	5.2 mmol/L^*^	3.5-5.1 mmol/L
Chloride	95 mmol/L^*^	98-107 mmol/L
Bicarbonate	22 mmol/L	20-30 mmol/L
Urea	20 mg/dL	10-20 mg/dL
Creatinine	0.75 mg/dL	0.57-1.11 mg/dL
Calcium (corrected for albumin)	9.2 mg/dL	8.4-10.8 mg/dL
Random glucose	387 mg/dL^*^	70-99 mg/dL
Liver function tests		
Aspartate aminotransferase (AST)	368 IU/L^*^	5-34 IU/L
Alanine aminotransferase (ALT)	161 IU/L^*^	11-55 IU/L
Alkaline phosphatase	105 IU/L	40-150 IU/L
Total bilirubin	0.5 mg/dL	0.2-1.2 mg/dL
Total protein	5.7 g/dL^*^	6.4-8.3 g/dL
Albumin	2.7 g/dL^*^	3.5-5.0 g/dL
Lactate dehydrogenase	788 IU/L^*^	125-220 IU/L
Creatinine kinase (CK)	15,069 IU/L^*^	29-168 IU/L
Lactic acid	2.8 mmol/L^*^	0.5-2.2 mmol/L
CRP	0.38 mg/dL	<0.90 mg/dL
Thyroid-stimulating hormone (TSH)	1.574 µIU/mL	0.350-4.940 µIU/mL

**Figure 4:**
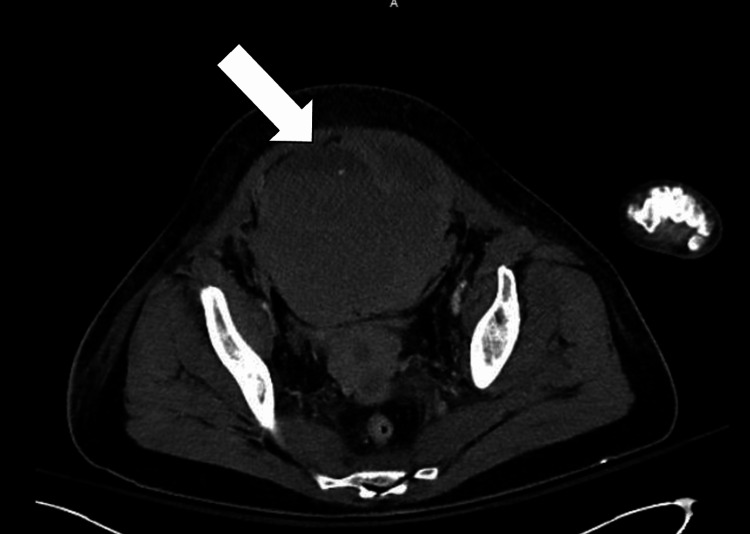
Computed tomography of the abdomen/pelvis with contrast. The image reveals a 12.8 x 11.8 cm left rectus sheath hematoma with evidence of subtle active extravasation above the urinary bladder and retroperitoneal inflammation/fluid (arrow).

On day three of her hospitalization, the patient acutely decompensated with hypotension, hypothermia, and acute blood loss anemia with diffuse abdominal pain and distention. Notable labs included hemoglobin 6.8 g/dL, hematocrit 19.1%, prothrombin time 19 s, partial prothrombin time 40 s, and an international normalized ratio of 1.3. Urgent CT abdomen/pelvis revealed a 12.8 x 11.8 cm left rectus sheath hematoma with evidence of subtle active extravasation (Figure 4). She was subsequently transferred to the intensive care unit due to concern for hemorrhagic shock and was initiated on norepinephrine for vasopressor support. Interventional radiology performed a pelvic arteriogram that demonstrated active extravasation from a branch of the left inferior epigastric artery for which they used gel foam and coil embolization to repair. The patient subsequently required increased vasopressor support with norepinephrine, vasopressin, epinephrine, and phenylephrine. She also underwent treatment with a massive transfusion protocol due to worsening anemia.

Initial myositis antibody panel, anti-neutrophil cytoplasmic antibody autoimmune panel, serum protein electrophoresis, urine protein electrophoresis, and porphyrins/porphobilinogen testing were unremarkable, refer to Table 3 for a breakdown of initial myositis panel reactivity.

**Table 3:** Initial myositis panel reactivity in a patient with dermatomyositis.

Parameter	Result	Reference range
Antinuclear antibody	Negative	Negative
Anti-double stranded DNA	2.1 IU/L	<9.9 IU/L
Anti-ribonuclear protein	<0.5 U/mL	<4.9 U/mL
Anti-Smith	<0.8 U/mL	<6.9 U/mL
Anti-Ro (SSA)	<0.4 U/mL	<6.9 U/mL
Anti-La (SSB)	<0.4 U/mL	<6.9 U/mL
SSA 52 (Ro) Ab, IgG	0 U/mL	0-40 U/mL
SSA 60 (Ro) Ab, IgG	0 U/mL	0-40 U/mL
Ribonucleic protein (U1) Ab, IgG	2 U/mL	0-19 U/mL
Antineutrophil cytoplasmic Ab, IgG	<1:20	<1:20
Jo-1 Ab, IgG	0 U/mL	0-40 U/mL
Myeloperoxidase Ab, IgG	1 U/mL	0-19 U/mL
Serine protease 3, IgG	0 U/mL	0-19 U/mL
Polymyositis_Scl 100 Ab, IgG	Negative	Negative
Signal recognition particle Ab	Negative	Negative
Anti-topoisomerase 1 (Scl-70)	<0.6 U/mL	<6.9 U/mL
Rheumatoid factor	<15 IU/mL	<30 IU/mL
Cyclic citrullinated peptide IgG	0.6 U/mL	<6.9 U/mL

**Figure 5:**
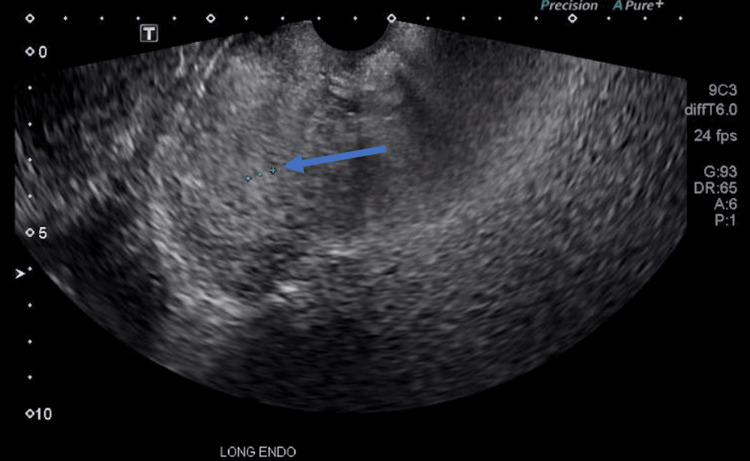
Transvaginal ultrasound demonstrating the long endometrial view of the uterus with an unremarkable endometrium measuring 7 mm in thickness (arrow).

Due to continued concern for underlying dermatomyositis and its well-known correlation with malignancy, further testing to rule out active neoplasm was completed. Our patient underwent a CT of the chest, abdomen, and pelvis which did not note any imaging findings concerning neoplasm. Specifically, the uterus was noted to be without significant abnormality. The patient reported that she had never undergone colonoscopy or mammogram screenings. Her last Pap smear was approximately eight years prior to presentation, with a history of one previous abnormal result requiring cold knife conization in her 20s. She did note irregular and infrequent menstrual cycles over the last 10 months, consistent with perimenopause. A transvaginal ultrasound was completed and demonstrated a mildly enlarged uterus with an unremarkable endometrium measuring 7 mm in thickness (Figure 5).

On hospital day six, our patient was able to be weaned from vasopressor support. Her blood counts stabilized without the need for continued transfusions. She continued to have worsening proximal muscle weakness, rash, and progressive dysphagia. On day 10 of her hospitalization high-dose corticosteroids were initiated due to continued concern for underlying dermatomyositis despite an initial negative myositis panel. The following day, the anti-nuclear matrix protein 2 (NXP-2) antibody returned as "high positive" from labs collected earlier in her hospitalization, refer to Table 4 for a breakdown of the extended myositis panel. The patient then underwent electromyography which demonstrated findings consistent with active myopathy.

**Table 4:** Extended myositis panel reactivity in a patient with dermatomyositis. ^*^Abnormal lab values.

Parameter	Result	Reference range
SAE1 (SUMO activating enzyme) Ab	Negative	Negative
NXP-2 (nuclear matrix protein) Ab	Highly positive^*^	Negative
MDA5 (CADM-140) Ab	Negative	Negative
Mi-2 (nuclear helicase protein) Ab	Negative	Negative
P155/140 Ab	Negative	Negative
PL-12 (alanyl-tRNA synthetase) Ab	Negative	Negative
PL-7 (threonyl-tRNA synthetase) Ab	Negative	Negative
OJ (isoleucyl-tRNA synthetase) Ab	Negative	Negative
RJ (glycyl-tRNA synthetase) Ab	Negative	Negative
Ku Ab	Negative	Negative
Fibrillarin (U3 RNP) Ab, IgG	Negative	Negative

**Figure 6:**
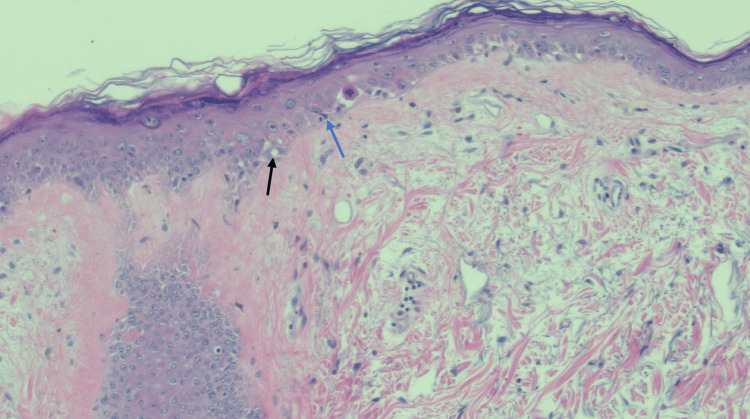
Hematoxylin and eosin staining (200x) of a skin punch biopsy of from the superior chest (V-sign area). The image demonstrates poikiloderma with epidermal atrophy, basal vacuolization (black arrow), and dyskeratotic keratinocytes with eosinophilic globules (blue arrow).

She was subsequently placed on prednisone 1 mg/kg daily for corticosteroid treatment. A skin biopsy of her erythematous rash was obtained and was reported as demonstrating sections of dermal atrophy, mid-basal vacuolar alteration, rare dyskeratotic cells, and mild perivascular inflammatory infiltrate consistent with connective tissue disorder such as dermatomyositis (Figure 6).

Our patient continued to have significant dysphagia despite corticosteroid treatment and ultimately required a percutaneous endoscopic gastrostomy (PEG) tube placement for continued enteral feeding. She was discharged 36 days after hospital admission with a plan to continue high-dose prednisone treatment with an extended steroid taper. She was also encouraged to obtain routine cancer screenings such as colonoscopy, mammogram, and Pap smear urgently in the outpatient setting.

**Figure 7:**
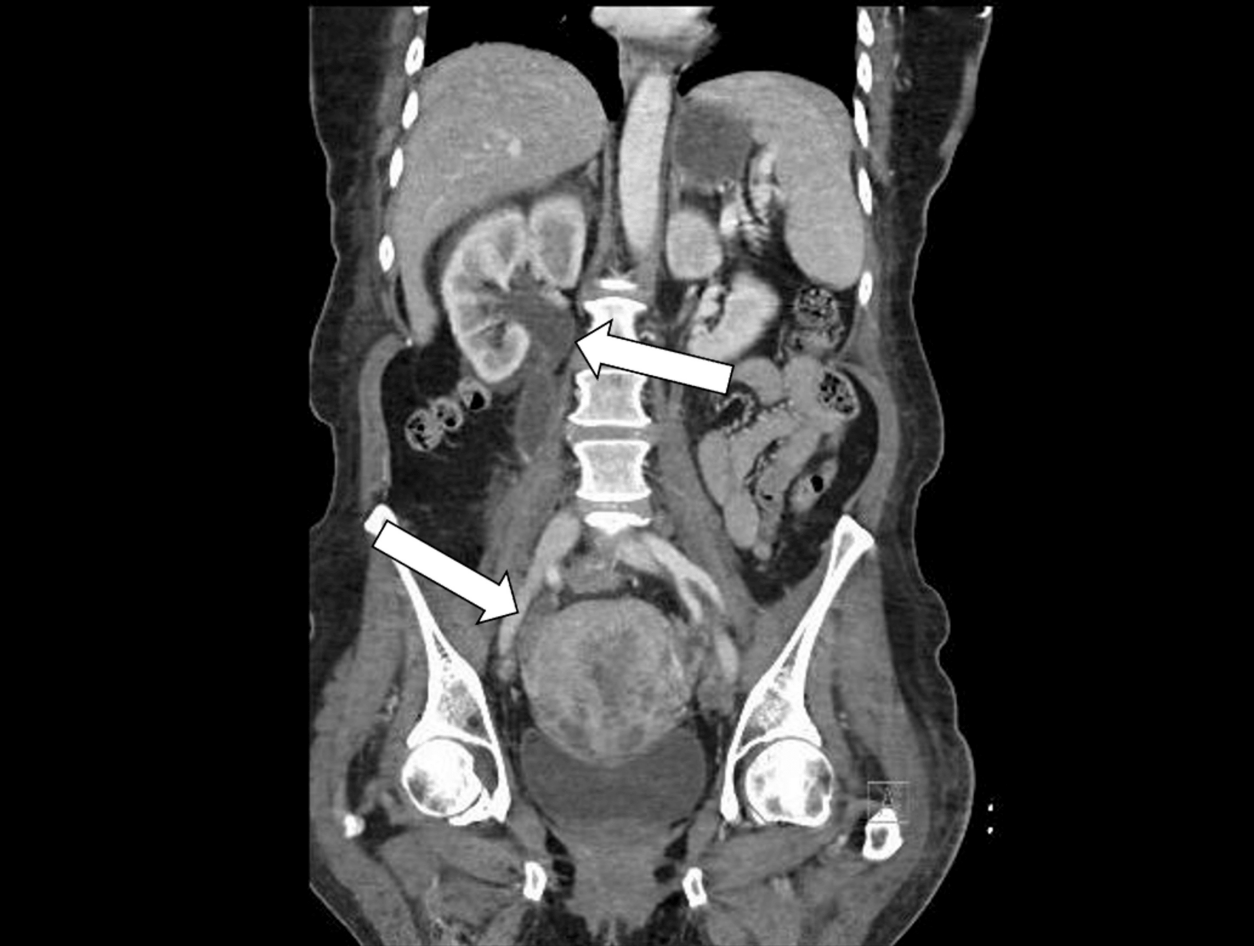
Computed tomography of the abdomen/pelvis with contrast. The image reveals right-sided hydronephrosis secondary to obstruction from nodular uterine mass (arrows).

Unfortunately, our patient was readmitted to the hospital almost 60 days after hospital discharge due to progressive abdominal pain and new-onset vaginal bleeding. She underwent a CT of the abdomen and pelvis and was found to have moderate right hydronephrosis due to distal ureteral obstruction from an occult tumor in this area. There was also a marked change in the uterus with multiple indistinct nodular enhancing lesions concerning neoplasm as well as a new periportal 2.6 cm hepatic hypodense lesion indeterminate for metastatic disease (Figure 7).

**Figure 8:**
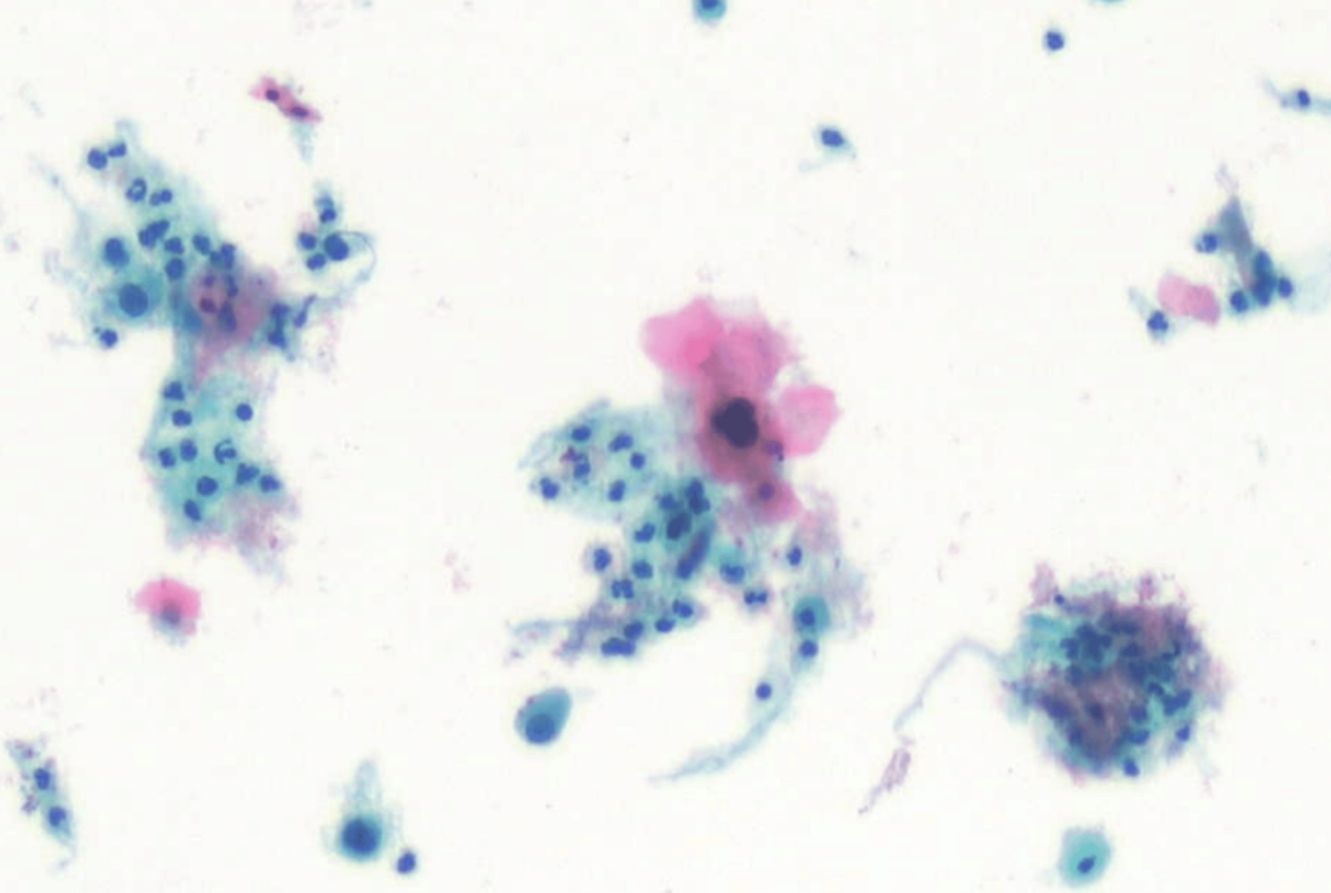
Pap smear cytology demonstrating malignant cells consistent with squamous cell carcinoma.

**Figure 9:**
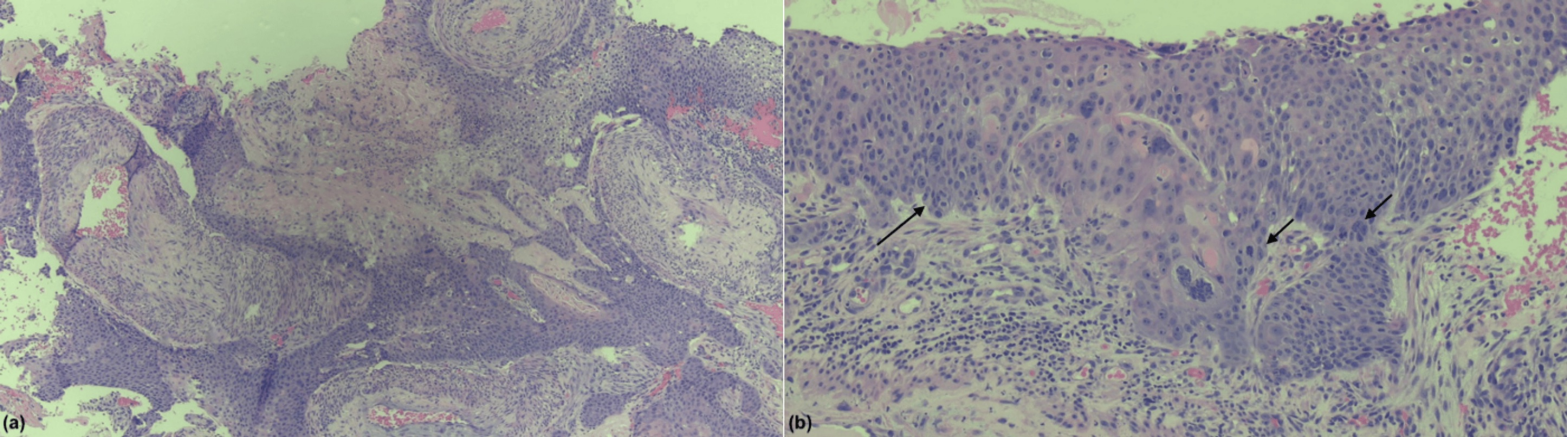
Hematoxylin and eosin staining of endometrial biopsy demonstrating moderately differentiated invasive squamous cell carcinoma. The images at (a) 40x magnification show invasion with abundant eosinophilic cytoplasm and keratinization and (b) 200x magnification show atypical cells (arrows) with enlarged nuclei and disorganized cell arrangement. Note: Pathology slide interpreted by Charles A. Mayfield.

**Figure 10:**
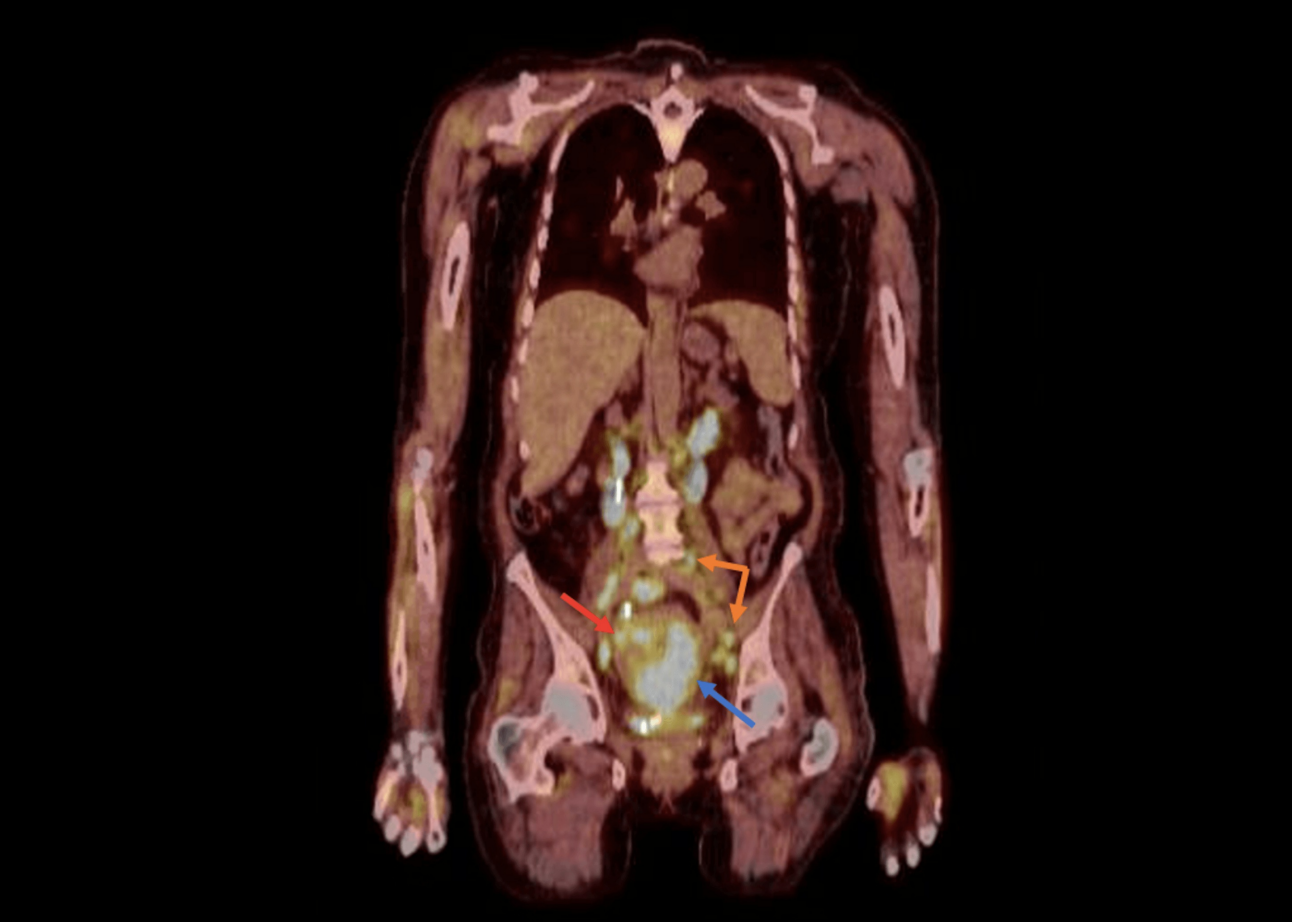
Positron emission tomography reveals a large primary carcinoma of the cervix (blue arrow) with extension to the upper uterus (red arrow) and multiple nodal metastases (orange arrows).

Due to these findings in combination with postmenopausal bleeding, our patient underwent a pelvic exam with a Pap smear and an endometrial biopsy. Pathology from these procedures demonstrated moderately differentiated, invasive squamous cell carcinoma of the cervix as well as evidence of positive high-risk human papillomavirus (Figures 8, 9a, 9b). She underwent a positron emission tomography (PET) scan just three months after her initial presentation to our hospital and normal CT imaging which revealed locally advanced primary carcinoma of the cervix, multiple abdominopelvic nodal metastases, and hypermetabolic right internal mammary and inferior left neck lymph nodes (Figure 10).

Staging at this time identified her as having stage IIIB squamous cell carcinoma of the cervix (T2bN1MX). She was recommended to undergo combination chemoradiation therapy. Throughout this time, our patient remained on prednisone therapy for treatment of her dermatomyositis. Unfortunately, our patient had rapid progression of her disease despite the treatment course described. She had multiple hospital admissions due to recurrent vaginal bleeding and eventual failure to thrive. Just six months after her initial presentation of dermatomyositis she succumbed to her disease and passed away with her family at her side.

## Discussion

Several forms of dermatomyositis are classified in the literature. The most common presentation is considered classic dermatomyositis and includes simultaneous muscle and cutaneous involvement as seen in our patient. Amyopathic dermatomyositis is a distinct variant in which patients lack myopathy or laboratory signs consistent with classic DM and instead present with only cutaneous involvement. Hypomyopathic dermatomyositis presents similarly to amyopathic DM with primarily cutaneous involvement; however, it does demonstrate laboratory evidence of underlying myositis. Juvenile dermatomyositis is an additional classification affecting children with a similar presentation as adult counterparts [1-3].

Several screening tests can be used to diagnose dermatomyositis. Some nonspecific screening tools include inflammatory markers such as erythrocyte sedimentation rate (ESR), C-reactive protein (CRP), and creatinine kinase. Other tests more commonly include myositis-specific antibodies such as anti-Jo-1, anti-Ro/SSA, anti-La/SSB, anti-smooth muscle, anti-ribonucleoprotein (RNP), anti-PM-Scl, and anti-Ku antibodies [4-6]. Of note, a negative routine myositis panel does not rule out a diagnosis of DM, as seen in our patient.

Cancer-associated myositis (CAM) is the term used to describe patients with DM who have developed malignancy [4,5]. The first case of inflammatory myopathy-related malignancy was documented in 1916 [7]. Since then time numerous studies have confirmed this association, more specifically in patients with DM [4-8]. Certain serum antibodies have been shown to correlate with an increased risk of malignancy whereas others have been shown to have a negative risk for the development of cancer. Autoantibodies that have been found to confer a positive risk for malignancy in CAM patients include antibodies to the transcription intermediary factor (TIF)-1 gamma (anti-p155, anti-p155/140) and antibodies to nuclear matrix protein-2 (NXP-2) [4,5,8,9]. Increased risk of malignancy has also been shown in patients with evidence of capillary damage on muscle biopsy, cutaneous necrosis, cutaneous vasculitis, older age at onset of DM, male sex, rapid onset of cutaneous manifestations/myalgias, and dysphagia [4,5,10-13]. Our patient had notable high positivity for the NXP-2 antibody in her extended myositis panel, rapid onset of symptoms, and evidence of progressive dysphagia requiring PEG tube placement indicating an increased risk of CAM.

With the well-defined risk of malignancy in patients with DM, the screening guidelines after diagnosis are not well-defined. Cancer can be diagnosed before, simultaneously with, or after the diagnosis of DM. Initial screening in all newly diagnosed patients includes complete blood count, liver function studies, urinalysis, chest radiograph, as well as age-appropriate fecal occult blood test, colonoscopy, mammography, and Pap testing [5,9]. Current guidelines suggest CT imaging only be performed if abnormal findings are found in these tests or for certain other findings associated with an increased risk of malignancy. These findings include older age of onset of DM, severe cutaneous disease, resistance to treatment, prior history of malignancy, absence of myositis-specific and myositis-associated antibodies, and the presence of p155/140 and/or anti-NXP2 antibodies [5,9].

Despite these screening recommendations, patients with DM have been shown to have an increased risk of cancer for at least five years after diagnosis, but this risk decreases annually [5]. Unfortunately, numerous studies have also found that CAM is associated with high all-cause mortality, such as in our patient [14].

The patient discussed in this case did have evidence of anti-NXP2 antibody reactivity warranting an initial CT scan for assessment. Screening with the described blood testing was able to be obtained during our patient’s initial hospitalization; however, outpatient testing with Pap smear, mammogram, and colonoscopy was deferred with the patient understanding to obtain these tests urgently. Due to the rapid development of invasive malignancy in our patient, this case raises the question of routine inpatient cancer screenings with a diagnosis of new-onset dermatomyositis.

## Conclusions

Dermatomyositis has a well-known association with malignancy. Malignancy screening has been proven to be invaluable and even lifesaving in certain cases of DM. This case demonstrates the importance of patient education about their elevated risk of malignancy that is associated with concurrent dermatomyositis, especially those with high-risk features, and brings to question the urgency of evaluation that is needed for cancer screening guidelines in these patients. All in all, for those with a complex pathology such as DM, malignancy screening should be a shared decision among the patient and a multidisciplinary team including their general practitioner, rheumatologist, and oncologist.
